# Inhibition of Monoacylglycerol Lipase by NSD1819 as an Effective Strategy for the Endocannabinoid System Modulation against Neuroinflammation-Related Disorders

**DOI:** 10.3390/ijms23158428

**Published:** 2022-07-29

**Authors:** Laura Micheli, Samuele Maramai, Alessandra Toti, Valentina Ferrara, Clara Ciampi, Lorenzo Di Cesare Mannelli, Carla Ghelardini

**Affiliations:** 1 Department of Neuroscience, Psychology, Drug Research and Child Health-Neurofarba-Pharmacology and Toxicology Section, University of Florence, Viale G. Pieraccini 6, 50139 Florence, Italy; alessandra.toti@unifi.it (A.T.); valentina.ferrara@unifi.it (V.F.); clara.ciampi@unifi.it (C.C.); lorenzo.mannelli@unifi.it (L.D.C.M.); carla.ghelardini@unifi.it (C.G.); 2 Department of Biotechnology, Chemistry and Pharmacy, University of Siena, Via Aldo Moro 2, 53100 Siena, Italy; maramai@unisi.it

**Keywords:** LPS, neuroinflammation, MGL inhibitor, glial cells, 2-AG, pain, depression, memory

## Abstract

Neuroinflammation is a key pathological event shared by different diseases affecting the nervous system. Since the underlying mechanism of neuroinflammation is a complex and multifaceted process, current pharmacological treatments are unsatisfactory—a reason why new therapeutic approaches are mandatory. In this context, the endocannabinoid system has proven to possess neuroprotective and immunomodulatory actions under neuroinflammatory status, and its modulation could represent a valuable approach to address different inflammatory processes. To this aim, we evaluated the efficacy of a repeated treatment with NSD1819, a potent β-lactam-based monoacylglycerol lipase inhibitor in a mouse model of neuroinflammation induced by lipopolysaccharide (LPS) injection. Mice were intraperitoneally injected with LPS 1 mg/kg for five consecutive days to induce systemic inflammation. Concurrently, NSD1819 (3 mg/kg) was daily per os administered from day 1 until the end of the experiment (day 11). Starting from day 8, behavioral measurements were performed to evaluate the effect of the treatment on cognitive impairments, allodynia, motor alterations, anhedonia, and depressive-like behaviors evoked by LPS. Histologically, glial analysis of the spinal cord was also performed. The administration of NSD1819 was able to completely counteract thermal and mechanical allodynia as highlighted by the Cold plate and von Frey tests, respectively, and to reduce motor impairments as demonstrated by the Rota rod test. Moreover, the compound was capable of neutralizing the memory loss in the Passive avoidance test, and reducing depressive-like behavior in the Porsolt test. Finally, LPS stimulation caused a significant glial cells activation in the dorsal horn of the lumbar spinal cord that was significantly recovered by NSD1819 repeated treatment. In conclusion, NSD1819 was able to thwart the plethora of symptoms evoked by LPS, thus representing a promising candidate for future applications in the context of neuroinflammation and related diseases.

## 1. Introduction

The term neuroinflammation broadly identifies the response of the central nervous system (CNS) against diseases or injuries. Despite neuroinflammation normally initiates as a defense response, acting with a protective goal, when it lasts for a longer time it can degenerate into a chronic immune system activation, leading to a pathological state. Indeed, it is well known that neuroinflammation is involved in the pathophysiology of several diseases affecting the nervous system such as psychiatric [1], immune [2], and neurodevelopmental disorders [3]. Moreover, neuroinflammation is implicated in the development and maintenance of chronic and neuropathic pain of different etiology, in which an excessive inflammation in the peripheral and/or central nervous system plays a pivotal role [4]. Lastly, recent works demonstrated how the coronavirus 2019 (COVID-19) infection triggered and reinforced obsessive and compulsive behavior, determining an aggravation of anxiety and depressive symptoms in patients affected by the virus [5,6,7]. The elevated levels of proinflammatory chemokines and cytokines and the consequent neuroinflammation evoked by the virus seems to be responsible for the pathophysiology of comorbid neuropsychiatric manifestations [8]. However, a dysregulation in neuroimmune mechanisms has been also detected in non-infected individuals that have been developed fatigue, brain fog, depression and other “sickness behavior”-like symptoms consequently to the pandemic crisis [9]. Neuroinflammation involves several cell types: microglia, oligodendrocytes, and astrocytes. Mast cells and microglia are the most important neuro-immune sentinels in the brain that cooperate with astrocytes to connect peripheral immune signaling with the CNS during inflammatory processes. All these cells are able to respond to harmful stimuli by producing chemokines and cytokines, fundamental for the pathological processes [10]. The lack of effective pharmacological treatments for neuroinflammation claims for the individuation of novel approaches that can prevent or ameliorate the multitude of diseases and symptoms that neuroinflammation can cause. 

**Figure 1:**
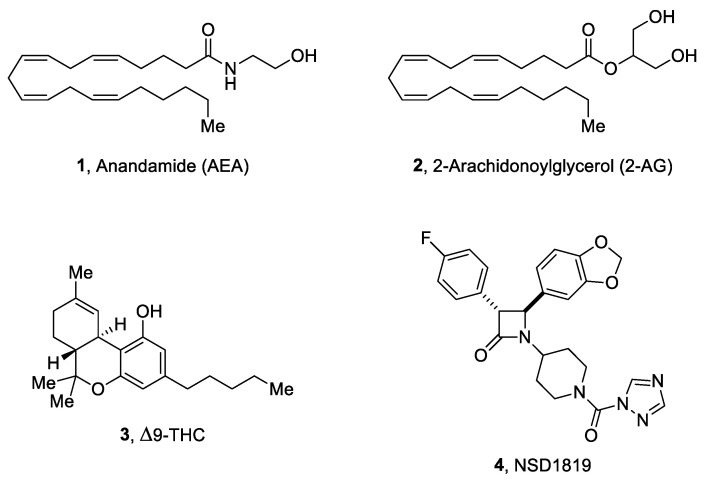
The structures of endocannabinoids AEA (**1**) and 2-AG (**2**), the natural cannabinoid Δ9-THC (**3**), and the title compound NSD1819 (**4**).

**Figure 2:**
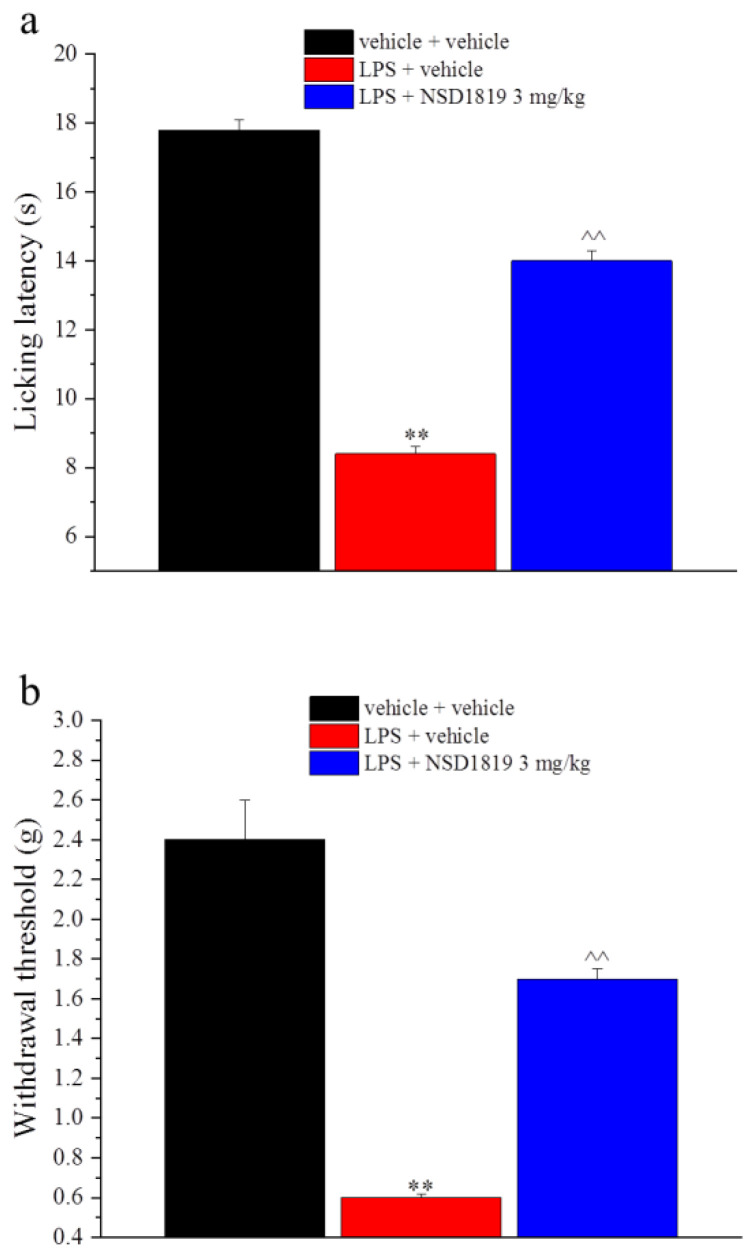
Effect of NSD1819 on LPS-induced thermal and mechanical allodynia. LPS (1 mg/kg) was intraperitoneally injected for five consecutive days (from 1 to 5). NSD1819 was suspended in 1% carboxymethylcellulose sodium salt (CMC) and daily orally administered starting from day 1 until the end of the experiment. Control animals were treated with vehicles. On day 8, 16 h after the last administration of the compound, (**a**) thermal and (**b**) mechanical allodynia were assessed by the Cold plate and von Frey tests, respectively. Data are expressed as the mean ± S.E.M. of values from 8 mice analyzed in 2 different experimental sets. ** *p* < 0.01 vs. vehicle + vehicle; ^^ *p* < 0.01 vs. LPS + vehicle.

In this context, the endocannabinoid system (ECS) resulted closely involved in different aspects of inflammation and immunomodulation. The ECS is composed of (i) the G-protein-coupled cannabinoid receptors (CBRs), classified as type 1 and type 2 cannabinoid receptors (CB1R and CB2R, respectively), along with the orphan receptor GPR55, which has been recently reported as a putative “type 3” CBR [11]; (ii) the endogenous ligands for CBRs, defined as endocannabinoids (ECB), and mainly represented by anandamide (AEA, **1**, Figure 1) and 2-arachidonylglycerol (2-AG, **2**, Figure 1); (iii) the enzymes responsible for the synthesis and catabolism of ECBs. Among these latter, the Fatty Acid Amide Hydrolase (FAAH) was originally identified as the enzyme responsible for AEA hydrolysis while the Monoacylglycerol Lipase (MGL) plays a pivotal role in the regulation of 2-AG levels in the CNS. [12,13]. Both CB1R and CB2R have been found to play an important part in the immunomodulatory processes. In fact, evidences from the literature demonstrated that administration of Δ9-tetrahydrocannabinol (THC, **3**, Figure 1) leads to apoptosis in T and dendritic cells, resulting in immunosuppression. In addition, several studies showed that cannabinoids downregulate cytokine and chemokine production with the suppression of the inflammatory responses [14]. CB1Rs are constitutively expressed on microglia while CB2Rs are expressed in activated cells but not in resting ones [15]. During the inflammation process, the whole ECS is upregulated to counteract the massive release of toxic mediators from microglia and consequently protect the cells from damage [16], suggesting that the modulation of the ECS represents a valid therapeutic strategy against neuroinflammation. While the administration of CBRs agonists may produce psychotropic effects through CB1R activation, thus limiting their use [17], the pharmacological inactivation of ECBs catabolic enzymes may constitute a valuable approach for ECS modulation [18,19,20]. 2-AG exerts several biological actions, modulating physiological processes like inflammation, memory, emotions, and pain [21,22]. Therefore, the use of compounds able to efficiently and selectively inhibit MGL, the main responsible for 2-AG hydrolysis in vivo, may be considered as a valid therapeutic option. In fact, this would be expected to elevate the endogenous concentrations of 2-AG and, consequently, to prolong and potentiate its beneficial effects, reducing the risk of the classical CBRs agonists’ side effects. Recently, a very potent and selective MGL inhibitor, namely NSD1819 (aka NF1819, **4**, Figure 1), has been reported [19]. NSD1819 is based on a *trans*-3,4-diaryl-substituted β-lactam scaffold and resulted to be a very potent inhibitor of *h*MGL (IC_50_ = 7.4 nM), acting through an irreversible mechanism of action, more than 380-fold selective over *h*FAAH and devoid of significant interaction with CB1R and CB2R (IC_50_ > 10 μM). The aim of this study was to evaluate the efficacy of a repeated treatment with NSD1819, in a mouse model of neuroinflammation reproduced by the repeated injection of lipopolysaccharide (LPS). Particularly, we analyzed the effectiveness of this compound against the plethora of symptoms that can occur during neuroinflammatory processes, such as memory impairment, depression, pain, and motor alterations. Moreover, ex vivo analyses were performed to determine the response of glial cells, the main actors in the pathophysiology of neuroinflammation, to the treatment with NSD1819.

### Chemistry 

**Scheme 1:**
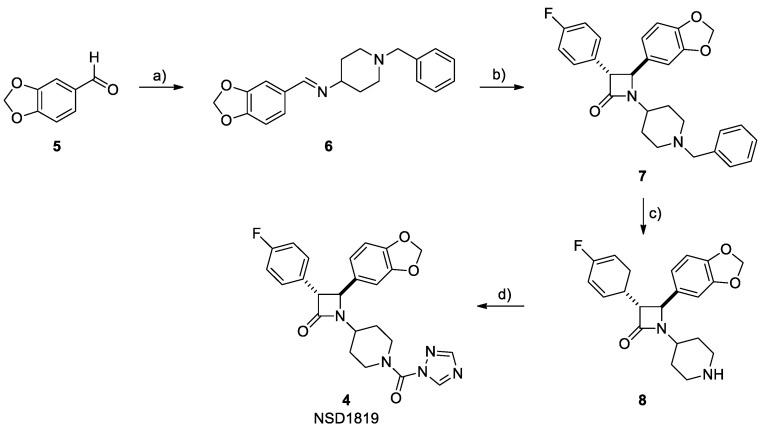
Reagents and conditions: (**a**) 4-amino-1-benzylpiperidine, EtOH, 80 °C, 2 h, quantitative yield; (**b**) triphosgene, dry DCM, 50 °C, 0.5 h then TEA, 50 °C, 12 h, 60%; (**c**) H_2_, Pd/C 10%, MeOH, 25 °C, 2 h, quantitative yield; (**d**) 1*H*-1,2,4-triazole, phosgene (20% in toluene), DMAP, dry DCM, 25 °C, 12 h, 63%.

NSD1819 (**4**, Figure 1) was efficiently synthesized following the originally reported procedure [19], here represented in Scheme 1. Briefly, 3,4-methylendioxy benzaldehyde (**5**) and 4-amino-1-benzylpiperidine were heated at reflux in absolute ethanol to afford the corresponding imine **6**. This latter was subjected to the Staudinger protocol, together with 4-fluorophenylacetic acid in the presence of triphosgene and TEA, achieving the β-lactam scaffold as a racemic mixture of the *trans*- configuration only. The benzyl group of intermediate **7** was then cleaved by means of a catalytic hydrogenation reaction, using 10% palladium on carbon as the catalyst, leading to the free amine **8**. The final reaction with 1*H*-1,2,4-triazole in the presence of phosgene and DMAP provided title compound **4**. 

## 2. Results

The mouse model of neuroinflammation was reproduced through the daily injection of LPS 1 mg/kg (for a total of 5 administrations, on days 1–5). NSD1819 3 mg/kg was administered daily per os from day 1 until the end of the behavioral experiments (day 11). From day 8, several symptoms of neuroinflammation were analyzed. As reported in Figure 2, LPS determined a significant decrease of the animals’ pain threshold, as measured by a thermal and mechanical non noxious stimuli (Cold plate and von Frey tests, respectively). The licking latency of the mouse significantly decreased from a value of about 18 s in the control group (vehicle + vehicle) to about 9 s in LPS + vehicle treated animals when the mice were exposed on a cold surface of 4 °C (Cold Plate test; Figure 2a). The daily treatment with NSD1819 was able to antagonize the thermal allodynia caused by the LPS repeated injections. It is important to remark that these results were obtained at least 16 h after the last administration of the compound and not immediately after the new daily treatment, which can correlate to a protective effect and not to a symptomatic one. Similarly, the von Frey test was used to measure the response of mice to a non-noxious mechanical paw stimulation. We observed a significant decrease of the withdrawal threshold in LPS-treated mice that was partially recovered by NSD1819 treatment (Figure 2b).

**Figure 3:**
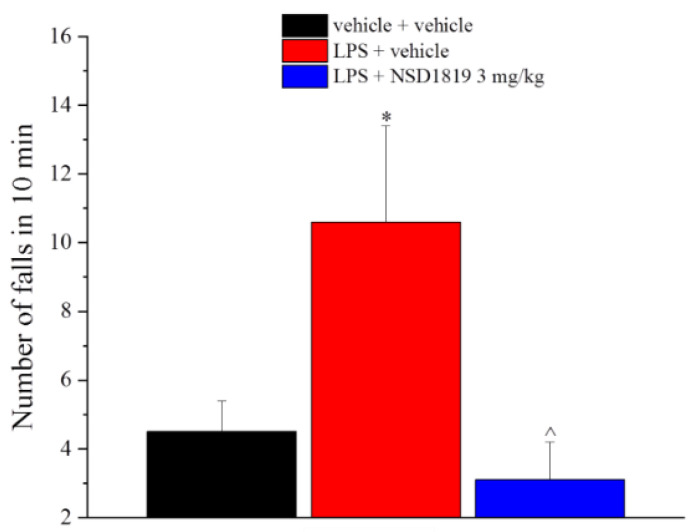
Effect of NSD1819 on LPS-induced motor alterations. LPS (1 mg/kg) was intraperitoneally injected for five consecutive days (from 1 to 5). NSD1819 was suspended in 1% carboxymethylcellulose sodium salt (CMC) and daily orally administered starting from day 1 until the end of the experiment. Control animals were treated with vehicles. On day 8, 16 h after the last administration of the compound, the motor coordination was assessed by the Rota rod test. Data are expressed as the mean ± S.E.M. of values from 8 mice analyzed in 2 different experimental sets. * *p* < 0.05 vs. vehicle + vehicle; ^ *p* < 0.05 vs. LPS + vehicle.

The LPS treatment also caused fatigue and motor impairment in mice evaluated by the Rota rod apparatus during 10 min of test session, in which a massive increase in the number of falls from the rod was highlighted in comparison to the control group (10.6 ± 2.8 vs. 4.5 ± 0.9, respectively). The daily treatment with NSD1819 improved the animal performance significantly, reducing the fatigue and the motor incoordination of mice that scored values comparable to those measured in the control group (Figure 3). 

The mouse spontaneous mobility and exploratory activity were also assessed by the hole-board test, as reported in Table 1. LPS did not alter these parameters in comparison to the control group, and no modifications were recorded in animals treated with the compound (Table 1).

**Figure 4:**
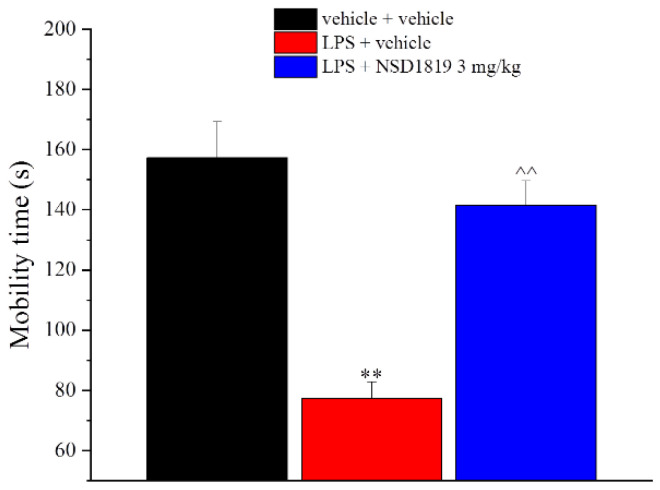
Effect of NSD1819 on LPS-induced depressive-like behavior. LPS (1 mg/kg) was intraperitoneally injected for five consecutive days (from 1 to 5). NSD1819 was suspended in 1% carboxymethylcellulose sodium salt (CMC) and daily orally administered starting from day 1 until the end of the experiment. Control animals were treated with vehicles. On day 8, 16 h after the last administration of the compound, the depressive-like behavior was assessed by the Porsolt test. Data are expressed as the mean ± S.E.M. of values from 8 mice analyzed in 2 different experimental sets. ** *p* < 0.01 vs. vehicle + vehicle; ^^ *p* < 0.01 vs. LPS + vehicle.

Neuroinflammation is also an important trigger for mood disorders and mnemonic deficits, reason why we assessed the efficacy of NSD1819 against depressive-like behavior by Porsolt test and against memory loss by the passive avoidance test. In the Porsolt test, where the mice were placed in a cylinder filled with water from which they cannot escape, LPS repeated administration determined a reduction of the mobility time in comparison to the control group (77.3 ± 5.6 s vs. 157.3 ± 12.0 s, respectively). This parameter was significantly increased by the treatment with NSD1819 that showed the capacity to protect the animals against depressive-like behavior evoked by the sub-chronic injection of LPS (Figure 4). 

**Figure 5:**
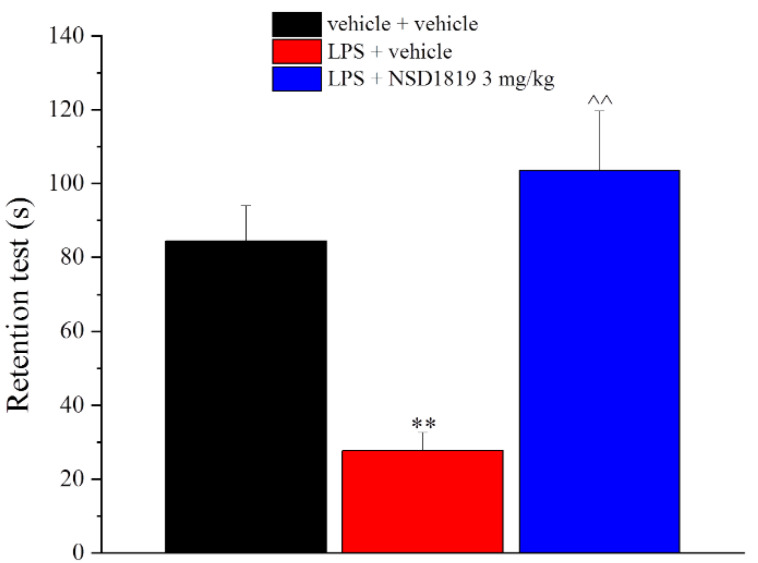
Effect of NSD1819 on LPS-induced memory loss. LPS (1 mg/kg) was intraperitoneally injected for five consecutive days (from 1 to 5). NSD1819 was suspended in 1% carboxymethylcellulose sodium salt (CMC) and daily orally administered starting from day 1 until the end of the experiment. Control animals were treated with vehicles. On day 8, 16 h after the last administration of the compound, the memory was assessed by the Passive avoidance test. Data are expressed as the mean ± S.E.M. of values from 8 mice analyzed in 2 different experimental sets. ** *p* < 0.01 vs. vehicle + vehicle; ^^ *p* < 0.01 vs. LPS + vehicle.

Moreover, the compound was challenged in reducing the amnesic effect of LPS in the Passive avoidance test (Figure 5). NSD1819 repeated treatment significantly antagonized the cognitive impairment induced by LPS. During the retention test, the time spent in the light box by the control group was 84.4 ± 9.7 s in comparison to 27.7 ± 4.9 s reached by LPS + vehicle group. LPS + NSD1819-treated mice prolonged the time spent in the light box up to 103.7 ± 16.0 s (Figure 5). 

**Figure 6:**
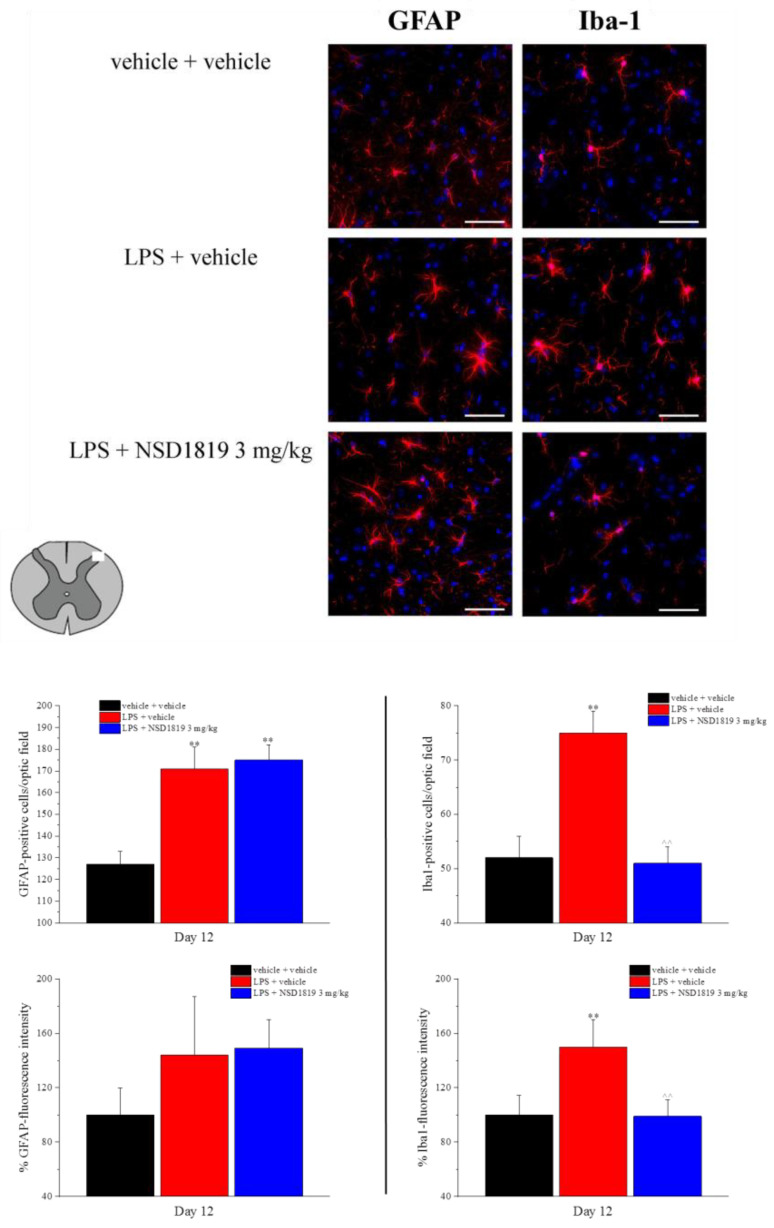
Effect of NSD1819 on LPS-induced glia cell activation in the dorsal horn of the lumbar spinal cord (L4–L5). LPS (1 mg/kg) was intraperitoneally injected for five consecutive days (from 1 to 5). NSD1819 was suspended in 1% carboxymethylcellulose sodium salt (CMC) and daily orally administered, starting from day 1 until the end of the experiment. Control animals were treated with vehicles. At the end of the behavioral experiments and 16 h after the last administration of the compound, animals were sacrificed, and the lumbar spinal cord was collected. GFAP and Iba-1 antibodies were used as marker for astrocytes and microglia, respectively. Representative images of merged GFAP-or Iba-1-labeled cells (red), plus DAPI-labeled cell nuclei (blue) at 40× magnification are shown (scale bar = 50 μm). Histograms show the quantitative analysis of GFAP and Iba-1 fluorescence intensity and the number of GFAP- and Iba-1 positive cells/optic field. Data are expressed as the mean ± S.E.M. of values from 8 mice analyzed in 2 different experimental sets. ** *p* < 0.01 vs. vehicle + vehicle; ^^ *p* < 0.01 vs. LPS + vehicle.

At the end of the behavioral measurements, the lumbar portion of the spinal cord was collected to analyze the glial cell profile in the dorsal horn by immunohistochemical analysis. Five LPS injections upregulated the number of GFAP and Iba1-positive cells as well as the Iba-1 fluorescence intensity (Figure 6). NSD1819 repeated treatment protected microglia from the maladaptive plasticity, but not the astrocytes. Indeed, the Iba-1 positive cells and the % of Iba-1 fluorescence intensity were significantly reduced in LPS + NSD1819 treated animals in comparison to the LPS + vehicle group (Figure 6). Nevertheless, the upregulation of GFAP-positive cells was not modified by the compound.

## 3. Discussion

Neuroinflammation is a process in reaction to a nervous system injury that begins as a protective response. Nevertheless, when the insult is prolonged it exceeds the bounds of physiological control, generating deleterious effects [23]. The reactive response of the CNS against elements that interfere with homeostasis is a common link between different diseases and emerging evidences suggest that understand and control inflammatory processes by guiding the interactions between the immune system and the nervous system may be the key for the prevention or treatment of most CNS pathologies [24]. The literature reported how a systemic infection can be linked to increased behavioral and cognitive complications [25]. Indeed, the stimulation of the peripheral immune system in mice causes exaggerated neuroinflammation, paralleled by depressive-like behaviors [26], prolonged sickness [27], and memory alterations [28]. Clinically speaking, the design of new therapeutics that, at the same time, can improve the recovery from symptoms, control the inflammatory processes, and modulate the immune response to attenuate the progression of the diseases, is therefore crucial. 

The ECS has been proposed as a promising candidate for the management of chronic neuroinflammation [29,30,31], although the molecular mechanisms that underlie these effects are still poorly understood [32]. However, it has been hypothesized that cannabinoids can regulate the pro-inflammatory responses by temper microglia activation, promoting their switch to the anti-inflammatory phenotype [33]. 

The ECS is a widespread neuromodulatory network that plays a central role in the regulation of many cognitive and physiological processes by modulating neuronal activity [34]. This system has been described as a relevant therapeutic target for different CNS disorders, particularly in neurodegenerative diseases such as Parkinson’s disease (PD), Alzheimer’s disease (AD), multiple sclerosis (MS), amyotrophic lateral sclerosis (ALS), pain, and epilepsy [35]. In fact, the endocannabinoid signaling seems to be altered and hypofunctional in many neurological diseases, thus representing a critical component in the control of neuroinflammation and the pathogenesis of disorders affecting the CNS. In this context, ECS modulators may serve as optimal pharmacological tools, and increasing attention has been paid to small molecules able to indirectly affect this system, trying to avoid the liabilities associated to typical direct agonists of CBRs, such as hypomotility, catalepsy or hypothermia. In fact, there is a crucial interplay between CBRs, ECBs, and their synthetic and metabolizing enzymes. The neuroprotective role of CBRs and their agonists has been already documented in neurodegenerative diseases [36]. Additionally, the non-psychoactive component of marijuana, the cannabidiol, showed neuroprotective activity [37]. Similarly, AEA and 2-AG exerted beneficial effects on inflammatory statuses and oxidative insults in a CBRs-mediated manner [38,39]. 2-AG is rapidly converted to arachidonic acid (AA) and glycerol by MGL, although multiple enzymes are involved in degradation of 2-AG, such as α/β-hydrolase domains 6 and 12 (ABHD6 and ABHD12, respectively) [40], leading to a decrease in 2-AG levels and signaling. In addition, it has been reported that the hydrolysis of 2-AG by MGL provides the major pool of AA for neuroinflammatory eicosanoids in specific tissues, such as brain, liver, and lungs [41]. The chemical inhibition or genetic disruption of MGL activity was able to lower LPS-induced pro-inflammatory eicosanoid production via a CB1 and CB2 receptor-independent mechanism [41]. By selectively inhibiting MGL, thus elevating the endogenous concentrations of 2-AG and indirectly activating CBRs, a great variety of positive effects on CNS and neuroinflammation can be achieved. In the present study, we highlight the efficacy of a NSD1819, a potent and selective MGL inhibitor, in a mouse model of neuroinflammation evoked by the sub-chronic injection of LPS. This agent was previously tested in a preclinical model of MS, namely mice suffering from experimental autoimmune encephalomyelitis (EAE), sharing some important features with the human disease. NSD1819 was able to remarkably influence the disease progression, as assessed by the significant lower daily clinical score observed in treated vs. vehicle injected EAE mice [13,19]. Our new investigation showed that the daily treatment with NSD1819 was able to counteract pain, motor incoordination and fatigue, depression, and memory impairment evoked by the above-mentioned polysaccharide. Moving to the ex vivo evaluation, NSD1819 demonstrated the capacity to reduce the activation of microglia that was upregulated by LPS in the dorsal horn of the spinal cord, counteracting the maladaptive plasticity that occurs to one of the main actors in neuroinflammation. In this work, the neuroinflammatory processes were evoked by the sub-chronic injection of LPS, a toll-like receptor 4 (TLR-4) ligand [42]. TLR-4 is primarily expressed in microglia in the CNS [43] and its activation determined the production of pro-inflammatory cytokines, prostaglandins, and NO [44,45], the key mediators for the neuroinflammatory processes. After four injections of LPS, mice reported mechanical and thermal allodynia as measured by the Von Frey and Cold plate tests, confirming that inflammation leads to the alteration of the pain threshold, inducing a pathological hypersensitivity, which determines the first way from physiological nociception to persistent pain [46]. The repeated treatment with NSD1819 counteracted the condition of hypersensitivity (Figure 2), allowing to hypothesize an effect mediated by the 2-AG-induced activation of CBRs, associated with a reduced pain sensation [47]. 

In fact, our MGL inhibitor was previously found to be significantly efficacious in another animal model of acute pain. Here, the levels of 2-AG in the spinal cord and paw skin of mice co-administered with formalin and NSD1819 were found to be elevated. The co-administration of CBRs antagonists significantly reduced this effect. Therefore, we concluded that NSD1819, by elevating endogenous 2-AG levels, was indirectly activating both CB1 and CB2 receptors, as previously reported for other MGL inhibitors [19]. This perfectly correlates with the fact that MGL represents the principal enzyme involved in 2-AG metabolism and its inhibition clearly influences 2-AG levels in vivo [48]. Knowing the mechanism of action of our compound, we concluded that NSD1819 clearly and positively influenced pain sensation in LPS-mice, and this effect was mediated by the modulation of the ECS.

The alteration of mice pain threshold was not the only symptom caused by LPS but also mood disorders like depression and anhedonia, as well as memory and motor impairments, were generated. Remarkably, all these alterations were counteracted by the treatment with NSD1819, with the exception of anhedonia (data not shown). 

In fact, the neuroinflammation associated with LPS treatment is responsible for the insurgence of mood disorders and other memory and motor deficits, without altering the spontaneous mobility and the exploratory activity of mice. NSD1819 was able to protect the animals against depressive-like behavior, as demonstrated by the Porsolt test, where a significant reduction of the mobility time in comparison to the control group was evidenced (Figure 4). In the passive avoidance test, representative of the amnesic effect induced by LPS, the cognitive deficits associated to the neuroinflammation were antagonized in NSD1819-treated animals (Figure 5). Fatigue and motor incoordination, substantially increased after LPS injection, were restored to levels comparable to the control group following NSD1819 administration in the Rota rod test (Figure 3). 

All these interesting effects induced by our compound were evaluated at least 16 h far from the last administration and not immediately after, thus suggesting a potential protective effect over a symptomatic one. To further confirm this hypothesis, we checked the activation of glial cells in LPS- and NSD1819-treated animals. Glial cells are an important cell population in the CNS, predominantly represented by microglia and astrocytes [49]. Among their functions there are the regulation of pH and ionic balance for the maintaining of homeostasis, uptake and degradation of neurotransmitters and modulation of neuroinflammation in physiological and pathological conditions [50,51]. Several studies pointed out the pivotal role of glial cells in the generation and maintenance of chronic pain [52,53] as well as neuropathic [54]. In response to cytokines and other signaling molecules from acute inflammation, microglia transform from a ramified, inactivated state to an activated phagocytic one, releasing pro-inflammatory mediators in the process. In terms of chronic neuroinflammation, these cells can remain activated for extended periods, releasing quantities of cytokines and neurotoxic molecules that contribute to long-term neurodegeneration [55]. Therefore, pharmacological strategies to suppress microglial and astrocytes activity are being explored as therapies demonstrating a valid option in the management of chronic and neuropathic pain states [54]. Although LPS may induce satellite glial cell activation in dorsal root ganglia [56], recent evidence suggests the importance of glial activation in the spinal cord [57,58]. In LPS-treated mice we recorded an increase in the number of glial fibrillary acidic protein (GFAP)-positive cells and up-regulation of GFAP expression as well as an increase in the number of Iba1-positive cells and up-regulation of Iba1 expression. The maladaptive plasticity of the microglia in the spinal cord was attenuated by the treatment with NSD1819, where the Iba-1 positive cells and the Iba-1 fluorescence intensity were significantly reduced in NSD1819 treated animals. All these evidences point out how the indirect stimulation of CBRs via MGL inhibition (linked to a potential increase of 2-AG levels) and the modulation of ECS can efficiently reduce LPS-induced inflammatory effects and counteract microglia activation, highlighting the importance of the inhibition of these cell population in the control of neuroinflammation.

## 4. Materials and Methods

### 4.1. Synthesis of NSD1819

All materials and reaction solvents were purchased from commercial suppliers and used without further purification. Reaction progress was monitored by TLC using silica gel 60 F254 (0.040−0.063 mm) with detection by UV. Silica gel 60 (0.040−0.063 mm) were used for column chromatography. ^1^H NMR and ^13^C NMR spectra were recorded on a Varian 300 MHz or Bruker 400 MHz and 600 MHz spectrometers by using the residual signal of the deuterated solvent as internal standard. Splitting patterns are described as singlet (s), doublet (d), triplet (t), quartet (q), quintet (p), and broad (br); the values of chemical shifts (δ) are given in ppm and coupling constants (J) in hertz (Hz). ESI-MS spectra were performed by an Agilent 1100 series LC/MSD spectrometer. Yields are referred to purified products and are not optimized. All moisture-sensitive reactions were performed under argon atmosphere using oven-dried glassware and anhydrous solvents. The chemical purity of NSD1819 (**4**) was determined using an Agilent 1260 Infinity instrument comprising a binary pump, an autosampler, an UV-DAD, and an ESI-MS detector. The chromatographic analysis was performed with an Agilent Poroshell 120 EC-C18 column (2.1 mm × 50 mm, 2.7 μm), injecting 2 μL of the sample. The analysis was carried out with a gradient elution, solvent A (water), and solvent B (acetonitrile), 95:5 to 5:95 over 15 min, at the flow rate of 0.4 mL/min and UV detector at 254 nm. The purity was ≥ 95.0%.

*N-(Benzo[d][1,3]dioxol-5-ylmethylene)-1-benzylpiperidin-4-amine* (**6**). A solution of 4-amino-1-benzylpiperidine (500 μL, 2.50 mmol) and (3,4-methylendioxy)benzaldehyde (5, 375 mg, 2.50 mmol) in absolute ethanol (30.0 mL) was heated at 80 °C for 2 h. The mixture was cooled to rt, and the solvent was removed under reduced pressure. The crude imine was used in the following step with no further purification (yield assumed quantitative). ESI-MS *m/z*: 323 [M+H]^+^, 345 [M+Na]^+^.

*trans-4-(Benzo[d][1,3]dioxol-5-yl)-1-(1-benzylpiperidin-4-yl)-3-(4-fluorophenyl)azetidin-2-one* (**7**). A solution of 4-fluorophenylacetic acid (580 mg, 3.75 mmol) and triphosgene (370 mg, 1.25 mmol) in dry dichloromethane (20.0 mL) was heated at 50 °C for 30 min. Then, a solution of imine 6 (2.50 mmol) in dry dichloromethane (10.0 mL) was added dropwise, followed by the addition of TEA (1.05 mL, 7.50 mmol), and the mixture was heated at 50 °C for 12 h. The solvent was removed under reduced pressure. The crude was purified by means of flash chromatography on silica gel (1:2 ethyl acetate/petroleum ether) to afford compound **7** (690 mg, 60% yield) as a pale-yellow oil. ^1^H NMR (300 MHz, CDCl_3_) δ 7.24 (m, 7H), 7.02 (t, *J* = 8.6 Hz, 2H), 6.89 (s, 1H), 6.81 (m, 2H), 5.98 (s, 2H), 4.37 (d, *J* = 2.2 Hz, 1H), 4.02 (d, *J* = 1.6 Hz, 1H), 3.58 (m, 1H), 3.44 (s, 2H), 2.83 (dd, *J* = 34.7, 11.2 Hz, 2H), 1.98 (m, 4H), 1.71 (dd, *J* = 12.6, 2.9 Hz, 1H), 1.52 (qd, *J* = 11.9, 3.9 Hz, 1H). ^13^C NMR (75 MHz, CDCl_3_) δ 168.4, 162.6 (d, *J*_C-F_ = 246.3 Hz), 148.7, 148.3, 138.4, 132.9, 131.3 (d, *J*_C-F_ = 3.2 Hz), 129.3, 129.2 (d, *J*_C-F_ = 8.1 Hz), 128.4, 127.3, 120.5, 116.1 (d, *J*_C-F_ = 21.5 Hz), 108.8, 106.4, 101.6, 63.9, 63.3, 63.2, 52.6, 52.40, 51.3, 30.7, 30.4. ESI-MS *m/z*: 459 [M+H]^+^, 481 [M+Na]^+^.

*trans-4-(Benzo[d][1,3]dioxol-5-yl)-3-(4-fluorophenyl)-1-(piperidin-4-yl)azetidin-2-one* (**8**). To a solution of **7** (250 mg, 0.55 mmol) in methanol (40.0 mL), a catalytic amount of Pd/C 10% was added, and the mixture was stirred under hydrogen atmosphere at 1 atm for 2 h. Next, Pd/C was filtered off, and the solvent was removed under reduced pressure. The crude transparent oil was used in the following step without any further purification (quantitative yield). ^1^H NMR (600 MHz, CD_3_OD) δ 7.30 (dd, *J* = 8.5, 5.3 Hz, 2H), 7.12 (t, *J* = 8.7 Hz, 2H), 7.03 (d, *J* = 1.3 Hz, 1H), 6.96 (dd, *J* = 7.9, 1.5 Hz, 1H), 6.87 (d, *J* = 7.9 Hz, 1H), 5.99 (s, 2H), 4.62 (d, *J* = 2.1 Hz, 1H), 4.22 (d, *J* = 1.6 Hz, 1H), 3.74–3.66 (m, 1H), 3.42 (dd, *J* = 9.5, 3.4 Hz, 2H), 3.35–3.32 (m, 4H), 3.07–2.96 (m, 2H), 2.25–2.15 (m, 2H), 2.11 (dd, *J* = 13.9, 2.9 Hz, 1H), 1.84 (qd, *J* = 11.9, 4.1 Hz, 1H). ^13^C NMR (75 MHz, CD_3_OD) δ 169.5, 162.6 (d, *J*_C-F_ = 245.1 Hz), 148.9, 148.6, 131.7, 131.0 (d, *J*_C-F_ = 3.2 Hz), 129.3 (d, *J*_C-F_ = 8.2 Hz), 120.1, 115.6 (d, *J*_C-F_ = 21.8 Hz), 108.5, 106.4, 101.7, 63.3, 42.8, 42.7, 27.1, 26.7. ESI-MS *m/z*: 369 [M + H]^+^. 

*trans-(1-(1-(1H-1,2,4-Triazole-1-carbonyl)piperidin-4-yl)-4-benzo[d][1,3]dioxol-5-yl)-3-(4-fluorophenyl)azetidin-2-one* (**4**). To a solution of 1*H*-1,2,4-triazole (15 mg, 0.22 mmol) in dry dichloromethane (10.0 mL), phosgene 20% solution in toluene (115 μL, 0.22 mmol) and DMAP (55 mg, 0.44 mmol) were added, and the mixture was stirred at 25 °C for 1 h. Next, a solution of amine **8** (40 mg, 0.11 mmol) in dry dichloromethane (5.0 mL) was added and the reaction was stirred at 25 °C for 12 h. The solvent was removed under reduced pressure. The crude was purified by means of chromatography on silica gel (1:1 ethyl acetate/n-hexane) to afford compound 4 (30 mg, 60% yield) as a white solid. ^1^H NMR (600 MHz, CDCl_3_) δ 8.76 (s, 1H), 7.98 (s, 1H), 7.22 (t, *J* = 6.2 Hz, 2H), 7.05 (t, *J* = 8.2 Hz, 2H), 6.89 (s, 1H), 6.86-6.83 (m, 2H), 6.02 (s, 2H), 4.70–4.41 (m, 2H), 4.39 (d, *J* = 2.3 Hz, H), 4.09 (d, *J* = 2.1 Hz, 1H), 3.78 (t, *J* = 10.5 Hz, 1H), 3.16 (dd, *J* = 28.7, 10.8 Hz, 2H), 2.23-2.01 (m, 2H), 1.91 (d, *J* = 12.8 Hz, 1H), 1.78–1.60 (m, 1H). ^13^C NMR (75 MHz, CDCl_3_) δ 168.4, 162.5 (d, *J*_C-F_ = 246.6 Hz), 152.3, 148.9, 148.6, 148.5, 146.9, 132.0, 130.8 (d, *J*_C-F_ = 3.3 Hz), 129.1 (d, *J*_C-F_ = 8.1 Hz), 120.6, 116.1 (d, *J*_C-F_ = 21.6 Hz), 108.9, 106.2, 101.7, 64.0, 63.4, 50.7, 45.5 (2C), 30.4, 30.1. LC-MS, RP water/acetonitrile 95:5 to 5:95, retention time: 7.35 min; ESI-MS *m/z*: 464 [M+H]^+^, 486 [M+Na]^+^.

### 4.2. Animals

For all the experiments described below, male CD-1 mice (Envigo, Varese, Italy) weighing approximately 25–30 g at the beginning of the experimental procedures were used. Animals were housed in CeSAL (Centro Stabulazione Animali da Laboratorio, University of Florence) and used at least one week after their arrival. Four rats were housed per cage (size 26 × 41 cm), kept at 23 ± 1 °C with a 12 h light/dark cycle, light at 7 a.m., and were fed a standard laboratory diet and tap water ad libitum.

### 4.3. LPS-Induced Neuroinflammation and NSD1819 Treatment

LPS (Sigma-Aldrich, St. Louis, MO, USA) 1 mg/kg was dissolved in sterile saline solution and intraperitoneally injected for 5 consecutive days from day 1 to day 5. NSD1819 [19] was suspended in 1% carboxymethylcellulose sodium salt (CMC) and daily per os administered from day 1 to day 11 at the dose of 3 mg/kg. Behavioral experiments were conducted from day 8 to day 12, always performed 16 h after the last administration of the compound. On day 12 the animals were sacrificed. Control animals were treated with vehicles.

### 4.4. Von Frey Test

The animals were placed in 20 × 20 cm Plexiglas boxes equipped with a metallic meshy floor, 20 cm above the bench. A habituation of 15 min was allowed before the test. An electronic von Frey hair unit (Ugo Basile, Varese, Italy) was used: the withdrawal threshold was evaluated by applying force ranging from 0 to 5 g with a 0.2 g accuracy. Punctuate stimulus was delivered to the mid-plantar area of each anterior paw from below the meshy floor through a plastic tip and the withdrawal threshold was automatically displayed on the screen.

The paw sensitivity threshold was defined as the minimum pressure required to elicit a robust and immediate withdrawal reflex of the paw. Voluntary movements associated with locomotion were not taken as a withdrawal response. Stimuli were applied on each anterior paw with an interval of 5 s. The measure was repeated 5 times, and the final value was obtained by averaging the 5 measures [59,60].

### 4.5. Cold Plate Test

Thermal allodynia was assessed using the cold plate test. With minimal animal–handler interaction, mice were taken from home-cages, and placed onto the surface of the cold plate (Ugo Basile, Varese, Italy) maintained at a constant temperature of 4 °C ± 1 °C. Ambulation was restricted by a cylindrical Plexiglas chamber (diameter, 10 cm; height, 15 cm), with open top. A timer controlled by foot peddle began timing response latency from the moment the mouse was placed onto the cold plate. Pain-related behavior (licking of the hind paw) was observed, and the time (seconds) of the first sign was recorded. The cutoff time of the latency of paw lifting or licking was set at 30 s [61,62]. 

### 4.6. Passive Avoidance Test

The test was performed according to the step-through method described by Jarvik and Kopp [63,64] with minor modification. The apparatus consisted of a two-compartment acrylic box with a lighted compartment connected to a darkened one by a guillotine door. In the original method, mice received a punishing electrical shock as soon as they entered the dark compartment, while in our modified method, after entry into the dark compartment, mice receive a non-painful punishment consisting of a fall (from 40 cm) into a cold-water bath (10 °C). For this purpose, the dark chamber was constructed with a pitfall floor. Mice receive the punishment when entering the dark room in the training session and remember it in the session on the following day, unless their memory is impaired. Latency times for entering the dark compartment were measured in the training test and after 24 h in the retention test. In the results section, the data recorded during the retention session are reported. The maximum entry latency allowed in the training and retention sessions was, respectively, 60 and 180 s. 

### 4.7. Porsolt Test

The forced swimming test used was the same described by Porsolt and colleagues [65]. Briefly, mice were placed individually into glass cylinders (height: 25 cm, diameter: 10 cm) containing 12 cm of water maintained at 22–23 °C and left there for 6 min. A mouse was judged to be immobile when it floated in the water, in an upright position, and made only small movements to keep its head above water. The duration of mobility was recorded during the last 4 min of the 6-min test. An increase in the duration of immobility is indicative of a depressant-like effect [66].

### 4.8. Locomotor Activity

The locomotor activity was evaluated by using the hole-board test. The apparatus consisted of a 40 cm square plane with 16 flush mounted cylindrical holes (3 cm diameter) distributed 4 × 4 in an equidistant, grid-like manner. Mice were placed on the center of the board one by one and allowed to move about freely for a period of 5 min each. Two photobeams, crossing the plane from mid-point to midpoint of opposite sides, thus dividing the plane into 4 equal quadrants, automatically signaled the movement of the animal (counts in 5 min) on the surface of the plane (locomotor activity). Miniature photoelectric cells, in each of the 16 holes, recorded (counts in 5 min) the exploration of the holes (exploratory activity) by the mice.

### 4.9. Rota Rod Test

The apparatus consisted of a base platform and a rotating rod with a diameter of 3 cm and a non-slippery surface. The rod was placed at a height of 15 cm from the base. The rod, 30 cm in length, was divided into 5 equal sections by 6 disks. Thus, up to five mice were tested simultaneously on the apparatus, with a rod-rotating speed of 16 revolutions per minute. The integrity of motor coordination was assessed on the basis of the number of falls from the rod in 10 min [67].

### 4.10. Tissue Collection and Immunohistochemistry of the Spinal Cord

On day 12, after the behavioral measurements, mice were sacrificed by decapitation and the lumbar spinal cord segments were removed, postfixed in 4% neutral buffered paraformaldehyde, and then cryoprotected in 30% sucrose solution at 4 °C. Slide-mounted cryostat sections (5 μm) were processed for indirect immunofluorescence histochemistry.

Formalin-fixed cryostat sections (5 μm) were incubated for 30 min in ready-to-go blocking solution (Bio-Optica, Milan, Italy) at room temperature to block unspecific binding. The primary antibodies, incubated overnight at 4 °C, were directed against Iba1 (rabbit, 1:200; Wako Chemicals, Richmond, VA, USA) for microglial staining or against glial fibrillary acidic protein (GFAP; rabbit, 1:200; Dako, Carpinteria, CA, USA) for astrocyte staining. After rinsing in PBST, sections were incubated in donkey anti-rabbit IgG secondary antibody labelled with Alexa Fluor 488 (1:500, Invitrogen, Milan, Italy) at room temperature for 1 h. Nuclei were stained with DAPI (4′,6-diamidin-2-fenilindolo; 1:2000; Invitrogen, Milan, Italy).

Negative control sections (no exposure to the primary antisera) were processed concurrently with the other sections for all immunohistochemical studies. We obtained a single optical density value for the dorsal horns by averaging the two sides in each mouse, and these values were compared to the homologous average values from the vehicle-treated animals.

Images were acquired by a motorized Leica DM6000B microscope equipped with a DFC350FX camera (Leica, Mannheim, Germany). 

Quantitative analysis of GFAP and Iba1-positive cells was performed by collecting at least three independent fields through a 40 × 0.5 NA objective. GFAP-positive cells were counted using the “cell counter” plugin of ImageJ, while Iba1-positive cells were quantified by means of the automatic thresholding and segmentation features of ImageJ. 

The mean fluorescence intensity of GFAP or Iba1 was calculated by subtracting the background (multiplied by the total area) from the GFAP or Iba1 integrated intensity. The GFAP or Iba-1 signal in immunostained sections was quantified using ImageJ software (NIH, Bethesda, MD, USA) by automatic thresholding images with the aid of the “triangles” algorithm, which we found to provide the most consistent pattern recognition across all acquired images. Analyses were performed on three different images for each animal, collected through a 40 × objective. Immunofluorescent staining was measured as mean fluorescence intensity using ImageJ software (ImageJ, National Institute of Health, USA, https://imagej.nih.gov/) by automatic thresholding algorithm. 

### 4.11. Statistical Analysis

Trained observers not informed about the specific treatment of each animal group carried out the tests. Results were expressed as means ± S.E.M. and the analysis of variance was performed by ANOVA test. A Bonferroni’s significant difference procedure was used as a post hoc comparison. *p* values less than 0.05 were considered significant. Data were analyzed using the ‘‘Origin 9.1′’ software.

## 5. Conclusions

The present work demonstrated the efficacy of a daily repeated treatment with NSD1819, a potent and selective inhibitor of MGL, in a mouse model of LPS-induced neuroinflammation. In particular, we contributed to further elucidate the involvement of the ECS in the regulation of mood disorders, pain, motor, and cognitive impairments related to neuroinflammation, and how blocking the catabolism of 2-AG can represent a valid strategy to counteract all these symptoms. Moreover, NSD1819 was able to reduce the maladaptive plasticity of microglia in the dorsal horn of the lumbar portion of the spinal cord. All the data collected confirm the ECS as a valuable therapeutic target for the management of neuroinflammation and highlight the efficacy of a small molecule modulator of 2-AG degradation, which deserves further attention and studies.

## Figures and Tables

**Table 1:** Hole board test.

Treatment	Board	Hole
vehicle + vehicle	70.4 ± 11.2	43.6 ± 6.0
LPS + vehicle	70.8 ± 6.7	41.9 ± 5.6
LPS + NSD1819 3 mg/kg	76.3 ± 7.7	39.0 ± 5.5

## Data Availability

Data are available upon reasonable request.

## References

[1] Radtke F.A., Chapman G., Hall J., Syed Y.A. (2017). Modulating Neuroinflammation to Treat Neuropsychiatric Disorders. BioMed Res. Int..

[2] Vezzani A. (2013). Fetal brain inflammation may prime hyperexcitability and behavioral dysfunction later in life: Vezzani: Fetal Brain Inflammation. Ann. Neurol..

[3] Bjørklund G., Saad K., Chirumbolo S., Kern J.K., Geier D.A., Geier M.R., Urbina M.A. (2016). Immune dysfunction and neuroinflammation in autism spectrum disorder. Acta Neurobiol. Exp..

[4] Ellis A., Bennett D.L.H. (2013). Neuroinflammation and the generation of neuropathic pain. Br. J. Anaesth..

[5] Rivera R.M., Carballea D. (2020). Coronavirus: A trigger for OCD and illness anxiety disorder?. Psychol. Trauma.

[6] Nissen J.B., Højgaard D.R.M.A., Thomsen P.H. (2020). The immediate effect of COVID-19 pandemic on children and adolescents with obsessive compulsive disorder. BMC Psychiatry.

[7] Nezgovorova V., Ferretti C.J., Pallanti S., Hollander E. (2021). Modulating neuroinflammation in COVID-19 patients with obsessive-compulsive disorder. J. Psychiatr. Res..

[8] Muccioli L., Pensato U., Bernabè G., Ferri L., Tappatà M., Volpi L., Cani I., Henry O.J., Ceccaroni F., Cevoli S. (2021). Intravenous immunoglobulin therapy in COVID-19-related encephalopathy. J. Neurol..

[9] Brusaferri L., Alshelh Z., Martins D., Kim M., Weerasekera A., Housman H., Morrissey E.J., Knight P.C., Castro-Blanco K.A., Albrecht D.S. (2022). The Pandemic Brain: Neuroinflammation in non-infected individuals during the COVID-19 pandemic. Brain, Behav. Immun..

[10] Skaper S.D. (2016). Mast Cell - Glia Dialogue in Chronic Pain and Neuropathic Pain: Blood-Brain Barrier Implications. CNS Neurol. Disord.—Drug Targets.

[11] Yang H., Zhou J., Lehmann C. (2016). GPR55—a putative “type 3” cannabinoid receptor in inflammation. J. Basic Clin. Physiol. Pharmacol..

[12] Lu H.-C., Mackie K. (2016). An Introduction to the Endogenous Cannabinoid System. Biol. Psychiatry.

[13] Maramai S., Brindisi M. (2020). Targeting Endocannabinoid Metabolism: An Arrow with Multiple Tips Against Multiple Sclerosis. ChemMedChem.

[14] Nagarkatti P., Pandey R., Rieder S.A., Hegde V.L., Nagarkatti M. (2009). Cannabinoids as novel anti-inflammatory drugs. Futur. Med. Chem..

[15] Cabral G.A., Raborn E.S., Griffin L., Dennis J., Marciano-Cabral F. (2008). CB2 receptors in the brain: Role in central immune function: Functional relevance of the CB2R in the CNS. Br. J. Pharmacol..

[16] Sánchez A.J., García-Merino A. (2012). Neuroprotective agents: Cannabinoids. Clin. Immunol..

[17] Knoller N., Levi L., Shoshan I., Reichenthal E., Razon N., Rappaport Z.H., Biegon A. (2002). Dexanabinol (HU-211) in the treatment of severe closed head injury: A randomized, placebo-controlled, phase II clinical trial*. Crit. Care Med..

[18] Butini S., Gemma S., Brindisi M., Maramai S., Minetti P., Celona D., Napolitano R., Borsini F., Cabri W., Fezza F. (2013). Identification of a novel arylpiperazine scaffold for fatty acid amide hydrolase inhibition with improved drug disposition properties. Bioorganic Med. Chem. Lett..

[19] Brindisi M., Maramai S., Gemma S., Brogi S., Grillo A., Di Cesare Mannelli L., Gabellieri E., Lamponi S., Saponara S., Gorelli B. (2016). Development and Pharmacological Characterization of Selective Blockers of 2-Arachidonoyl Glycerol Degradation with Efficacy in Rodent Models of Multiple Sclerosis and Pain. J. Med. Chem..

[20] Brindisi M., Borrelli G., Brogi S., Grillo A., Maramai S., Paolino M., Benedusi M., Pecorelli A., Valacchi G., Di Cesare Mannelli L. (2018). Development of Potent Inhibitors of Fatty Acid Amide Hydrolase Useful for the Treatment of Neuropathic Pain. ChemMedChem.

[21] Mackie K. (2008). Cannabinoid Receptors: Where They are and What They do. J. Neuroendocr..

[22] Alhouayek M., Masquelier J., Muccioli G.G. (2014). Controlling 2-arachidonoylglycerol metabolism as an anti-inflammatory strategy. Drug Discov. Today.

[23] Disabato D.J., Quan N., Godbout J.P. (2016). Neuroinflammation: The devil is in the details. J. Neurochem..

[24] Morales I., Farías G.A., Cortes N., Maccioni R.B., Moretti D.V. (2016). Neuroinflammation and Neurodegeneration. Update on Dementia.

[25] Asby D., Boche D., Allan S., Love S., Miners J.S. (2021). Systemic infection exacerbates cerebrovascular dysfunction in Alzheimer’s disease. Brain.

[26] Godbout J.P., Moreau M., Lestage J., Chen J., Sparkman N.L., Connor J.O., Castanon N., Kelley K.W., Dantzer R., Johnson R.W. (2008). Aging Exacerbates Depressive-like Behavior in Mice in Response to Activation of the Peripheral Innate Immune System. Neuropsychopharmacology.

[27] Godbout J.P., Chen J., Abraham J., Richwine A.F., Berg B.M., Kelley K.W., Johnson R.W. (2005). Exaggerated neuroinflammation and sickness behavior in aged mice after activation of the peripheral innate immune system. FASEB J..

[28] Chen J., Buchanan J.B., Sparkman N.L., Godbout J.P., Freund G.G., Johnson R.W. (2008). Neuroinflammation and disruption in working memory in aged mice after acute stimulation of the peripheral innate immune system. Brain Behav. Immun..

[29] Pacher P., Bátkai S., Kunos G. (2006). The Endocannabinoid System as an Emerging Target of Pharmacotherapy. Pharmacol. Rev..

[30] Ashton J.C., Glass M. (2007). The Cannabinoid CB2 Receptor as a Target for Inflammation-Dependent Neurodegeneration. Curr. Neuropharmacol..

[31] Saito V.M., Rezende R.M., Teixeira A.L. (2012). Cannabinoid Modulation of Neuroinflammatory Disorders. Curr. Neuropharmacol..

[32] Tanaka M., Sackett S., Zhang Y. (2020). Endocannabinoid Modulation of Microglial Phenotypes in Neuropathology. Front. Neurol..

[33] Young A.P., Denovan-Wright E.M. (2022). The Dynamic Role of Microglia and the Endocannabinoid System in Neuroinflammation. Front. Pharmacol..

[34] Szabo B., Schlicker E., Pertwee R.G. (2005). Effects of Cannabinoids on Neurotransmission. Cannabinoids.

[35] Basavarajappa B.S., Shivakumar M., Joshi V., Subbanna S. (2017). Endocannabinoid system in neurodegenerative disorders. J. Neurochem..

[36] Chung Y.C., Bok E., Huh S.H., Park J.-Y., Yoon S.-H., Kim S.R., Kim Y.-S., Maeng S., Park S.H., Jin B.K. (2011). Cannabinoid Receptor Type 1 Protects Nigrostriatal Dopaminergic Neurons against MPTP Neurotoxicity by Inhibiting Microglial Activation. J. Immunol..

[37] Hampson A.J., Grimaldi M., Axelrod J., Wink D. (1998). Cannabidiol and (−)Δ ^9^ -tetrahydrocannabinol are neuroprotective antioxidants. Proc. Natl. Acad. Sci. USA.

[38] Jia J., Ma L., Wu M., Zhang L., Zhang X., Zhai Q., Jiang T., Wang Q., Xiong L. (2014). Anandamide Protects HT22 Cells Exposed to Hydrogen Peroxide by Inhibiting CB1 Receptor-Mediated Type 2 NADPH Oxidase. Oxidative Med. Cell. Longev..

[39] Lourbopoulos A., Grigoriadis N., Lagoudaki R., Touloumi O., Polyzoidou E., Mavromatis I., Tascos N., Breuer A., Ovadia H., Karussis D. (2011). Administration of 2-arachidonoylglycerol ameliorates both acute and chronic experimental autoimmune encephalomyelitis. Brain Res..

[40] Li W., Blankman J.L., Cravatt B.F. (2007). A Functional Proteomic Strategy to Discover Inhibitors for Uncharacterized Hydrolases. J. Am. Chem. Soc..

[41] Baggelaar M.P., Maccarrone M., van der Stelt M. (2018). 2-Arachidonoylglycerol: A signaling lipid with manifold actions in the brain. Prog. Lipid Res..

[42] Beutler B. (2000). Tlr4: Central component of the sole mammalian LPS sensor. Curr. Opin. Immunol..

[43] Lehnardt S., Lachance C., Patrizi S., Lefebvre S., Follett P.L., Jensen F.E., Rosenberg P.A., Volpe J.J., Vartanian T. (2002). The Toll-Like Receptor TLR4 Is Necessary for Lipopolysaccharide-Induced Oligodendrocyte Injury in the CNS. J. Neurosci..

[44] McGeer P.L., McGeer E.G., Yasojima K., Jellinger K., Schmidt R., Windisch M. (2000). Alzheimer Disease and Neuroinflammation. Advances in Dementia Research.

[45] Mrak R.E., Griffin W.S.T. (2005). Glia and their cytokines in progression of neurodegeneration. Neurobiol. Aging.

[46] Hsieh C.-T., Lee Y.-J., Dai X., Ojeda N.B., Lee H.J., Tien L.-T., Fan L.-W. (2018). Systemic Lipopolysaccharide-Induced Pain Sensitivity and Spinal Inflammation Were Reduced by Minocycline in Neonatal Rats. Int. J. Mol. Sci..

[47] Calignano A., La Rana G., Giuffrida A., Piomelli D. (1998). Control of pain initiation by endogenous cannabinoids. Nature.

[48] Hohmann A.G., Suplita R.L., Bolton N.M., Neely M.H., Fegley D., Mangieri R., Krey J.F., Walker J.M., Holmes P.V., Crystal J.D. (2005). An endocannabinoid mechanism for stress-induced analgesia. Nature.

[49] Jha M.K., Suk K. (2013). Glia-based biomarkers and their functional role in the CNS. Expert Rev. Proteom..

[50] Gwak Y.S., Kang J., Unabia G.C., Hulsebosch C.E. (2012). Spatial and temporal activation of spinal glial cells: Role of gliopathy in central neuropathic pain following spinal cord injury in rats. Exp. Neurol..

[51] Parpura V., Heneka M.T., Montana V., Oliet S.H.R., Schousboe A., Haydon P.G., Stout R.F., Spray D.C., Reichenbach A., Pannicke T. (2012). Glial cells in (patho)physiology: Glial cells in (patho)physiology. J. Neurochem..

[52] Watkins L.R., Maier S.F. (2002). Beyond Neurons: Evidence That Immune and Glial Cells Contribute to Pathological Pain States. Physiol. Rev..

[53] Barragán-Iglesias P., Pineda-Farias J.B., Cervantes-Durán C., Bravo-Hernández M., Rocha-González H.I., Murbartián J., Granados-Soto V. (2014). Role of Spinal P2Y_6_ and P2Y_11_ Receptors in Neuropathic Pain in Rats: Possible Involvement of Glial Cells. Mol. Pain.

[54] Di Cesare Mannelli L., Pacini A., Micheli L., Tani A., Zanardelli M., Ghelardini C. (2014). Glial role in oxaliplatin-induced neuropathic pain. Exp. Neurol..

[55] Liu B., Gao H.-M., Hong J.-S. (2003). Parkinson’s disease and exposure to infectious agents and pesticides and the occurrence of brain injuries: Role of neuroinflammation. Environ. Health Perspect..

[56] Blum E., Procacci P., Conte V., Hanani M. (2014). Systemic inflammation alters satellite glial cell function and structure. A possible contribution to pain. Neuroscience.

[57] Cao F.-L., Xu M., Wang Y., Gong K.-R., Zhang J.-T. (2015). Tanshinone IIA attenuates neuropathic pain via inhibiting glial activation and immune response. Pharmacol. Biochem. Behav..

[58] Silva G.D., Lopes P.S., Fonoff E.T., Pagano R.L. (2015). The spinal anti-inflammatory mechanism of motor cortex stimulation: Cause of success and refractoriness in neuropathic pain?. J. Neuroinflammation.

[59] Sakurai M., Egashira N., Kawashiri T., Yano T., Ikesue H., Oishi R. (2009). Oxaliplatin-induced neuropathy in the rat: Involvement of oxalate in cold hyperalgesia but not mechanical allodynia. Pain.

[60] Micheli L., Durante M., Lucarini E., Sgambellone S., Lucarini L., Di Cesare Mannelli L., Ghelardini C., Masini E. (2021). The Histamine H_4_ Receptor Participates in the Anti-Neuropathic Effect of the Adenosine A_3_ Receptor Agonist IB-MECA: Role of CD4^+^ T Cells. Biomolecules.

[61] Baptista-De-Souza D., Di Cesare Mannelli L., Zanardelli M., Micheli L., Nunes-De-Souza R.L., Canto-De-Souza A., Ghelardini C. (2014). Serotonergic modulation in neuropathy induced by oxaliplatin: Effect on the 5HT2C receptor. Eur. J. Pharmacol..

[62] Micheli L., Di Cesare Mannelli L., Del Bello F., Giannella M., Piergentili A., Quaglia W., Carrino D., Pacini A., Ghelardini C. (2020). The Use of the Selective Imidazoline I1 Receptor Agonist Carbophenyline as a Strategy for Neuropathic Pain Relief: Preclinical Evaluation in a Mouse Model of Oxaliplatin-Induced Neurotoxicity. Neurotherapeutics.

[63] Jarvik M.E., Kopp R. (1967). An Improved One-Trial Passive Avoidance Learning Situation. Psychol. Rep..

[64] Micheli L., Di Cesare Mannelli L., Lucarini E., Parisio C., Toti A., Fiorentino B., Rigamonti M.A., Calosi L., Ghelardini C. (2020). Intranasal Low-Dose Naltrexone Against Opioid Side Effects: A Preclinical Study. Front. Pharmacol..

[65] Porsolt R.D., Le Pichon M., Jalfre M. (1977). Depression: A new animal model sensitive to antidepressant treatments. Nature.

[66] Micheli L., Spitoni S., Di Cesare Mannelli L., Bilia A.R., Ghelardini C., Pallanti S. (2020). Bacopa monnieri as augmentation therapy in the treatment of anhedonia, preclinical and clinical evaluation. Phytother. Res..

[67] Di Cesare Mannelli L., Maresca M., Micheli L., Farina C., Scherz M.W., Ghelardini C. (2017). A rat model of FOLFOX-induced neuropathy: Effects of oral dimiracetam in comparison with duloxetine and pregabalin. Cancer Chemother. Pharmacol..

[68] McGrath J.C., Lilley E. (2015). Implementing guidelines on reporting research using animals (ARRIVE etc.): New requirements for publication in BJP. J. Cereb. Blood Flow Metab..

